# Metagenome-assembled genomes of bacterial communities in the eastern Southwest Indian Ridge, Indian Ocean

**DOI:** 10.1128/mra.00870-25

**Published:** 2026-01-09

**Authors:** T. Jabir, S. Venkatachalam, L. Surya Prakash

**Affiliations:** 1 National Centre for Polar and Ocean Research, Ministry of Earth Sciences, Headland Sada, Vasco da Gama, Goa, India; Montana State University, Bozeman, Montana, USA

**Keywords:** Indian Ocean Ridges, eSWIR, hydrothermal plumes, metabolic functions

## Abstract

This paper presents high-quality metagenome-assembled genomes (MAGs) recovered from hydrothermal plume at the 67.67°E vent field along the eastern Southwest Indian Ridge. A total of 24 high-quality MAGs were obtained having 21 different genera. These MAGs, associated with chemosynthetic pathways including methane, metal, and sulfur metabolism, offer crucial insights into microbial transformation in deep-sea hydrothermal vents.

## ANNOUNCEMENT

Hydrothermal vents along the mid-ocean ridge provide energy in the form of chemical compounds that support chemolithoautotrophic microorganisms. The chemical composition of hydrothermal fluids is controlled by water-rock reactions. In the Indian Ocean, several vent fields have been reported along the Carlsberg, Central Indian, Southeast, and Southwest Indian Ridges. Previous reports on turbidity (1) and dissolved methane concentrations along the eastern Southwest Indian Ridge (eSWIR: 63.5°E–68°E) indicated hydrothermal plumes at several locations. A hydrothermal field near 67.67°E showed methane concentrations up to 37.8 nmol/kg in the plume, and the stable carbon isotope ratios of methane showed evidence of methane oxidation by microbes (2).

During the expedition onboard MGS Sagar in 2017, water-column studies were carried out in the eSWIR for the identification of hydrothermal plumes. A Sea Bird conductivity, temperature, depth (CTD) rosette equipped 24 Niskin bottles was used for sample collection. Ten liters of seawater was filtered through 0.22 µm polycarbonate membrane filters (Merck-Millipore). The filters collected were immediately frozen at −80°C onboard ship. The UltraClean Soil DNA Isolation Kit (MOBIO, CA) was utilized for DNA extraction. The quality of DNA was checked by Qubit fluorometry using the Qubit dsDNA Assay Kit (Invitrogen, USA). The metagenomic shotgun library prepared from DNA samples from each station using the NEBNext Ultra DNA Library Prep Kit (New England Biolabs). The libraries were screened through Tapestation (Agilent Technologies) and sequenced using paired-end sequencing (2×150 bp) chemistry on an Illumina HiSeq X10 platform. The sequencing yielded approximately 8–19 million reads among the samples (Table 1).

**TABLE 1:** Information of metagenome samples for generating the MAGs and their SRA accession details and assembly statistics

	Sample name	Bio project number	SRA accession	No. of Illumina reads	Source	Depth	Latitude	Longitude	Metagenome assembly statistics			
									Total length	N50	GC (%)	Number of contigs
1	30B	PRJNA1290625	SRX29647450	8,117,606	Bottom water	3,770	26° 96 S	66° 81 E	84,053,159	10,507	54.09	18,187
2	54B	PRJNA1290625	SRX29647451	9,860,009	Bottom water	4,777	27° 91 S	64° 45 E	104,724,730	8,326	54.1	26,524
3	58P	PRJNA1290625	SRX29647452	10,045,489	Plume layer	3,920	26° 57 S	67° 75 E	49,544,529	4,290	54.16	162,320
4	68B	PRJNA1290625	SRX29647453	19,423,976	Bottom water	3,980	27° 75 S	63° 80 E	92,301,340	17,499	52.26	17,648

The raw read sequences were assessed for quality using FastQC v0.12.0 (3). Low-quality sequences and adapters were removed by Illumina-utils and Anvio v7.1 (4). The reads were assembled into contigs using metaSPAdes v3.15.2 (5). The quality of assembly was assessed using MetaQUAST v5.0.2 (6). MAGs reconstruction from metagenomic assemblies was achieved using a reference-based workflow within Anvi'o (7). This process included contig database generation, ORF prediction with Prodigal v2.6.2 (8), annotation of contigs for single-copy core genes, and sequence coverage determination using Bowtie2 v2.3.5 and SAM tools v1.15.1 (7, 9). Coverage and detection statistics were generated using the anvi-profile algorithm (10, 11). Automated binning was conducted using CONCOCT v1.1.0, MaxBin2 v2.2.7, and MetaBAT2 v2.12.1 (10, 12, 13). The MAGs were refined manually using Anvio’s interactive interface and assessed for quality. The high-quality MAGs were sorted with the parameters of >90% completeness and <5% redundancy. The taxonomic identification of MAGs was carried out using the GTDB v1.5.1 database (14).

**Fig 1:**
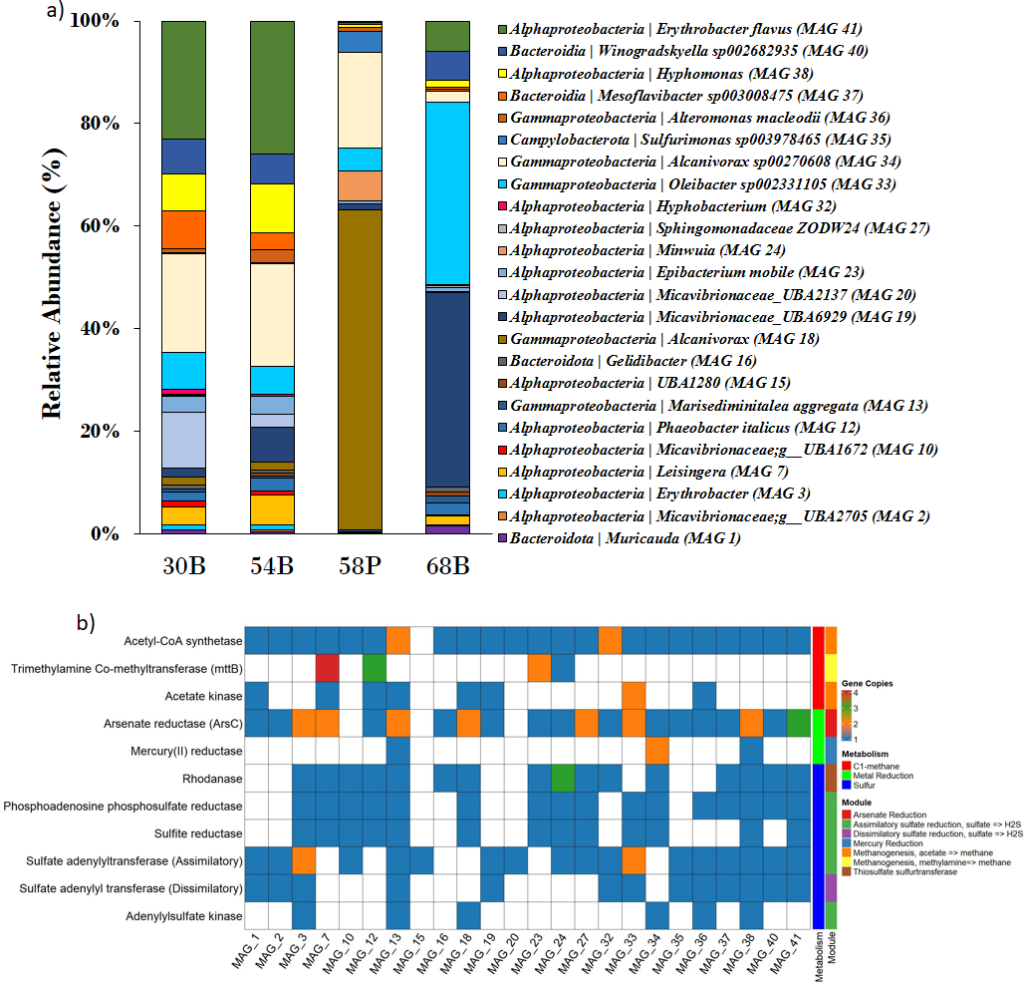
Details of relative abundance, taxonomy, and major functional annotation of MAGs. (**a**) Taxonomy and relative abundance of high-quality MAGs. (**b**) Functional annotations of the high-quality MAGs related to methane, sulfur, and metal metabolism.

The study identified 24 high-quality MAGs, with *Alcanovarox* sp. showing the highest abundance (~60%, Fig. 1a) in the hydrothermal plume layer (67.67°E vent field). Functional annotations of the MAGs showed the genes related to methanogenesis, assimilatory and dissimilatory sulfur, arsenic, and mercury reduction metabolisms (Fig. 1b). The mercury and arsenic reduction genes were previously reported from eSWIR (15). The identified metabolic pathways are known to significantly influence plume geochemistry by transforming dissolved gases and metals, thereby establishing a direct link between biological activity and elemental fluxes in the deep sea (16, 17). Thus, the present data on MAGs support the role of microbes in changing the chemical composition of hydrothermal plumes.

## Data Availability

The metagenome-assembled genome data were deposited in the NCBI database under BioProject PRJNA1290625. BioSample accession numbers for the raw reads and genomes are SAMN49923560 to SAMN49923563. MAG sequences, supplementary table, and further metadata are available in the open repository Figshare (https://doi.org/10.6084/m9.figshare.29820410).
